# Predictors of Classes of Well-Being Among Aging Women

**DOI:** 10.1093/geroni/igab046.1819

**Published:** 2021-12-17

**Authors:** Eileen Rillamas-Sun, Barbara Cochrane, Kenneth Pike, Nancy Woods

**Affiliations:** 1 Fred Hutchinson Cancer Research Center, Seattle, Washington, United States; 2 University of Washington, Seattle, Washington, United States

## Abstract

Our aim was to examine the relationship of predictors of well-being from prior studies to the well-being profile developed from data from aging WHI participants. Class 1 included women with both low hedonic and eudaemonic well-being scores, class 4 with the highest scores. Classes 2 and 3 had moderate scores, with class 2 having higher hedonic and lower eudaemonic scores and class 3 having lower hedonic and higher eudaemonic scores. We examined associations between predictors and well-being classes. Youngest women were in Class 4 (mean=60.2 years) and oldest in Class 3 (mean=63.2). African American women had higher proportions in in Classes 2 and 3, Latinas in Classes 1 and 3, and Asian/Pacific Islanders in Class 3. College graduates, married women and those with household incomes >$50,000 were most likely in Class 4. Associations with age, race/ethnicity, education, marital status and income were consistent with prior analyses incorporating individual well-being indicators.

